# Site of Tracheostomy and Its Influence on The Surgical Outcome and Quality of Life After Tracheal Resection and Anastomosis in Patients with Tracheal Stenosis

**DOI:** 10.1055/s-0043-1776702

**Published:** 2024-01-04

**Authors:** Soorya Pradeep, Arun Alexander, Sivaraman Ganesan, Dharanya Gopalakrishnan Srinivasan, Akshat Kushwaha, Aparna Gopalakrishnan, Lokesh Kumar Penubarthi, Kalaiarasi Raja, Sunil Kumar Saxena

**Affiliations:** 1 Department of ENT, Christian Medical College (CMC), Vellore, India; 2 Department of ENT, Jawaharlal Institute of Postgraduate Medical Education & Research (JIPMER), Pondicherry, India

**Keywords:** trachea, tracheostomy, dyspnea, airway management

## Abstract

**Introduction** With the advances in critical care, the incidence of post intubation tracheal stenosis is increasing. Tracheal resection and anastomosis have been the gold standard for the management of grades III and IV stenosis. Scientific evidence from the literature on the determining factors and outcomes of surgery is not well described.

**Objective** This study was aimed at determining the influence of tracheostoma site on the surgical outcomes and postoperative quality of life of patients undergoing tracheal resection anastomosis.

**Methods** Thirteen patients who underwent tracheal resection and anastomosis during a period of 3 years were followed up prospectively for 3 months to determine the degree of improvement in their quality of life postsurgery by comparing the pre and postoperative validated Tamil/vernacular version of RAND SF-36 scores and Medical Research Council (MRC) dyspnea score.

**Results** As per preoperative computed tomography (CT), the mean length of stenosis was found to be 1.5 cm while the mean length of trachea resected was 4.75 cm. We achieved a decannulation rate of 61.53%. There was an estimated loss of 3.20 +/− 1.90 cm of normal trachea from the lower border of the stenosis until the lower border of the stoma that was lost during resection.

Analysis of SF-36 and MRC dyspnea scores revealed significant improvement in the domains of physical function postoperatively in comparison with the preoperative scores ( *p* < 0.05).

**Conclusion** Diligent placement of tracheostomy in an emergency setting with respect to the stenotic segment plays a pivotal role in minimizing the length of the resected segment of normal trachea.

## Introduction

With the advances in critical care, the incidence of tracheal stenosis is increasing, with the predominant etiology being intubation-related injury. 1 A spectrum of interventions has been described for the management of these cases ranging from endoscopic interventions to open surgical techniques. The pros and cons of each have been studied in detail with newer technology being incorporated to minimize morbidity and mortality.

Although a variety of newer modalities of treatment have been described, tracheal resection and anastomosis has been the gold standard for the management of grades III and IV stenosis (Meyer and Cotton grades of stenosis) since its inception in the latter half of the 20th century. 2

While a plethora of data is available regarding the predictors of surgical success, improvisation of surgical techniques, anticipated complications and success rates, very few studies are available which view the determining factors and outcomes from the patients' perspective. 3 4 5 6 7 8

Though widely considered a safe procedure with minimal mortality and morbidity, it remains an intricate, invasive procedure which has a major impact on the patients' quality of life (QoL). The incidence of risks is minimal if literature is to be believed, but the risks have far-reaching consequences as far as the individual patient is concerned. In this era of litigation, it is necessary that we have an evidence base to counsel patients regarding the surgery so that they have an adequate understanding as to the risks and benefits. 3 9 10 11 12 13 14

Hence, it becomes imperative that we have evidence-based guidelines regarding patient selection and surgical outcomes so that both the patient and the surgeon have pragmatic expectations regarding the surgical outcome.

The present study was done to analyze the key determining factors that had an impact on surgery as well as the outcomes and QoL in patients with grades III and IV tracheal stenosis post tracheal resection and anastomosis by determining decannulation rates and QoL, with the RAND SF-36-item short form health survey instrument (validated Tamil version) (SF-36) and the Medical Research Council (MRC) dyspnea score. We have also presented the pivotal lessons that were of paramount importance in the management of tracheal stenosis.

## Materials and Methods

### Study Design

A prospective observational study was done on all patients above the age of 18 years who presented to the ENT OPD in JIPMER between the period of 2017 to 2019 with grades III to IV tracheal stenosis and were candidates for tracheal resection and anastomosis. Patients with long-segment stenosis (> 4 rings) or stenosis of the thoracic trachea requiring a sternotomy and patients with concomitant respiratory or cardiac illness were excluded.

All patients were either tracheostomized at the time of presentation or they presented in stridor and underwent an emergency tracheostomy. In all 13 cases who were recruited in this study, the tracheostoma was created with mere visualization of the closest tracheal segment possible below the level of lower border of the stenotic segment under dire emergency conditions.

Informed consent was obtained from all the patients who participated in the study. Approval from the Institute Research Council and Ethics Committee (JIP/IEC/2017/0355) was obtained. All provisions of the Declaration of Helsinki were followed. Preoperative quality of life was determined by administering a validated Tamil version of the SF-36 questionnaire ( Supplementary Material File 1 ) and MRC dyspnea scale. Preoperative QoL was defined as the QoL of the patient prior to tracheostomy.

### Sample Size

The study of Elsayed et al. 15 observed that decannulation rate was 96.7%. Taking this value as reference, the minimum required sample size with 10% margin of error and 5% level of significance is 13 patients. So, the total sample size taken is 13.

Formula used is: -

N ≥ (p [1 - p])/(ME/zα)2

Where Zα is the value of Z at two-sided alpha error of 5%, ME is margin of error, and p is proportion of patients with decannulation.

Calculations:

N ≥ ([0.967 * (1 - 0.967]) / (0.1/1.96) 2 = 12.26 = 13 (approx.)

The data collected included parameters such as patients' age, indication and duration of intubation, the interval between extubation and onset of symptoms and any interventions undergone prior to resection and anastomosis.

All patients were subjected to preoperative laryngotracheal endoscopic assessment to determine the grade of the stenotic segment (Meyer-Cotton classification), the position of the stenotic segment, the mobility of the vocal cords and the status of the infrastomal trachea (inflammation, granulations, tracheomalacia) and the number of healthy rings present infrastomally until the carina.

All patients underwent routine preoperative investigations (blood investigations, chest x-ray, electrocardiogram [ECG], echocardiogram [ECHO]) for . They also underwent non-contrast computed tomography (CT) of the neck and thorax to determine the diameter (dSS) and the length (lSS) of the stenotic segment, the distance from the lower border of the cricoid to the upper border of the stenotic segment, and the length of segment to be resected (loTR), that is, the length of the trachea from the upper border of stenosis till the lower end of the tracheostoma measured along the longitudinal axis of the trachea.

### Methodology

Patients were counselled regarding the surgery and its potential risks and benefits, and informed consent was obtained in writing. Consent was also taken for intraoperative photography and videography.

Patients underwent segmental tracheal resection with primary anastomosis according to the technique described by Grillo and Pearson et al. 3 4 Anastomosis was aided by laryngeal release maneuvers, such as infrahyoid release and blunt dissection of the lower trachea up to mediastinum. The course in the hospital, including the duration of hospital stay and complications encountered, were documented. Complications were managed as per departmental protocol, including revision tracheostomy in case of severe stridor. Patients were followed up with clinical evaluation and endoscopic assessment at 1 and 3 months postoperatively and as and when indicated. Patients underwent flexible bronchoscopic assessment at 3 weeks and rigid endoscopic assessment under general anesthesia at 6 weeks. Any granulations or stenosis noted were managed with debridement or endoscopic dilatation as required. At the end of 3 months, the SF-36 questionnaire was re-administered, and the MRC dyspnea score was calculated to determine improvement in the QoL postsurgery. Patient complaints such as dyspnea, dysphagia, poor voice quality, aspiration, and any postoperative interventions required were also noted.

### Statistical Analysis

The presentation of the categorical variables was done in the form of number and percentage (%). On the other hand, the quantitative data with normal distribution were presented as the mean ± SD and the data with non-normal distribution as median with 25th and 75th percentiles (interquartile range). The data normality was checked by using the Kolmogorov-Smirnov test. For those cases in which the data was not normal, we used nonparametric tests. The comparison of emotional wellbeing and general health between pre and postoperative was analyzed using a paired t-test and MRC dyspnea, whereas physical function, role limitation due to physical health, role limitation due to emotional problems, fatigue, social functioning, and bodily pain were analyzed using the Wilcoxon signed rank test. An independent t-test was used to associate length of stenosis (cm), minimum diameter of stenotic segment (cm), and preoperative estimation of loTR (cm), and the Mann-Whitney test was used for association of distance from cricoid (cm) with decannulation. Univariate logistic regression was used to find out OR with 95% CI.

The data entry was done in the Microsoft EXCEL spreadsheet (Microsoft Corp., Redmond, WA, USA), and the final analysis was done with the use of SPSS Statistics for Windows, version 17.0 software (SPSS Inc., Chicago, IL, USA.

For statistical significance, a *p* -value < 0.05 was considered statistically significant.

## Results

Of the 13 patients, 8 were male and 5 were female. The patients ranged from 19 to 36 years of age with a median age of 24 years. Occupationally, most of them were students or housewives. ( Table 1 )

**Table 1:** Distribution of demographic and clinical characteristics of study subjects

Demographic and clinical characteristics	Frequency	Percentage
**Age(years)**		
Mean ± SD	23.69 ± 4.9	
Median (25th–75th percentile)	24(20–25)	
Range	19–36	
**Gender**		
Female	5	38.46%
Male	8	61.54%
**Occupation**		
Housewife	2	15.38%
Laborer	1	7.69%
Mason	1	7.69%
Mechanic	2	15.38%
Student	7	53.85%

All the patients developed tracheal stenosis secondary to intubation for various indications, with the predominant indication being suicidal organophosphate poisoning (n = 9). Other indications included snake bite (n = 2), scorpion sting (n = 1), and road traffic accident (n = 1). The mean duration of intubation was 12.5 +/− 4.58 days.

The patients were tracheostomized for prolonged intubation in order to wean them off ventilatory support. They were asymptomatic for a period of 11.42 +/− 9.54 days before the onset of dyspnea and stridor. One patient experienced only minimal breathlessness on exertion for 15 years, which progressed to frank stridor.

As per the preoperative CTs, the mean length of stenosis was 1.68 +/− 1.09 cm, extending to 0.62 +/− 1.29 cm from the lower border of the cricoid. The mean diameter of the narrowest portion of the stenosis was 0.4 +/− 0.30 cm. In all 13 patients described in this series, the site of the tracheostoma was below the level of the lower border of the stenotic segment. We estimated the mean loTR (the length from the upper border of the stenotic segment to lower border of the tracheostoma measured along the axis of the trachea) as 4.75 +/− 1.41 cm. Hence, there was an estimated loss of 3.20 +/− 1.90 cm of normal trachea from the lower border of the stenosis until the lower border of the stoma that was lost during resection. Since the tracheostomy was made below the level of the stenotic segment, the stenotic segment was entirely included in resection ( Table 2 )

**Table 2:** Descriptive statistics of computed tomography parameters of study subjects

CT parameters	Mean ± SD	Median (25th–75th percentile)	Range
Length of stenosis (cm)	1.68 ± 1.09	1.4(0.9–2)	0.8–4.8
Minimum diameter of stenotic segment (cm)	0.4 ± 0.3	0.5(0–0.6)	0–0.8
Distance from cricoid (cm)	0.75 ± 1.28	0.9(0.6–1.36)	–2.97–2.32
Preoperative estimation of length of trachea to be resected (cm)	4.75 ± 1.41	4.82 (3.57–5.7)	2.59–7.04

Abbreviations: CT, computed tomography; SD, standard deviation.

The preoperative endoscopic assessment showed that the maximum number of patients had grade-III stenosis (grade III: IV = 10:3) and that, infrastomally, 10 healthy rings were present (median of 10 with a range of 8–14). The vocal cords were mobile in all the patients assessed. Two patients underwent endoscopic dilatation prior to resection and anastomosis.

We achieved a decannulation rate of 61.53% (n = 8). Postoperative interventions in the form of endoscopic dilatation were necessary due to excessive granulation at the anastomotic site in 28.57% (n = 4) patients, with 1 patient requiring 5 dilatations and the rest only 1.

Two patients required revision tracheostomy for restenosis.

Two patients could not be weaned off T-tube (15%). In one case, the decision to resect and perform anastomosis was discarded in favor of tracheoplasty, and the trachea was closed over a T-tube. In another instance, the patient could not be extubated postoperatively, and the endotracheal tube got blocked with secretions necessitating emergency tracheostomy on POD-1. Unfortunately, during the process of securing the airway, a 3-cm rent was created in the posterior tracheal wall. The patient underwent emergency exploration, and the trachea was closed over a T-tube.

One case had to be abandoned without attempting resection. Intraoperatively, we found that there was suprastomal collapse, and retraction had resulted in the trachea being pulled up leading to the assumption that there was an adequate length of trachea above the sternal notch. We discovered that further mobilization of the cervical trachea was not possible without a sternotomy, and the case had to be abandoned.

There was no significant association of CT parameters (length of stenosis, diameter of stenotic segment, distance from cricoid, and LoTR) with success of decannulation. ( Table 3 )

**Table 3:** Association of computed tomography parameters with decannulation

CT parameters	Decannulation (n = 8)	No decannulation (n = 5)	Total	*P* -value	Odds ratio (95% CI)
Length of stenosis(cm)	1.81 ± 1.3	1.48 ± 0.74	1.68 ± 1.09	0.621 *	1.178 (0.387–3.587)
Minimum diameter of stenotic segment (cm)	0.43 ± 0.29	0.36 ± 0.36	0.4 ± 0.3	0.724 *	1.934 (0.042–88.436)
Distance from cricoid (cm)	0.85 (0.507-1.212)	1.1 (0.7-1.36)	0.9 (0.6-1.36)	0.77 †	0.877 (0.34–2.261)
Preoperative estimation of length of trachea to be resected(cm)	4.57 ± 1.1	5.03 ± 1.92	4.75 ± 1.41	0.592 *	0.813 (0.354–1.866)

Abbreviations: CI, confidence interval; CT, computed tomography.

* Independent t test, ^†^ Mann Whitney test

Patient responses to the SF-36 questionnaire were recoded from 0 to 100, and the means were calculated to determine the domain scores for each patient. Analysis of SF-36 scores as per the Wilcoxon signed-rank test in patients with successful anastomosis revealed that the domains of physical function and general health showed significant improvement postoperatively ( *p* < 0.05). MRC dyspnea scores also showed significant improvement in comparison with the preoperative scores ( *p* < 0.05). ( Table 4 )

**Table 4:** Comparison of preoperative and postoperative Medical Research Council dyspnea and its components of study subjects

MRC dyspnea and its components	Mean ± SD	Median (25th–75th percentile)	Range	*P* -value
**MRC dyspnea**				
Preoperative	3.62 ± 1.19	4 (2.75–4.25)	2–5	0.016 §
Postoperative	1.5 ± 0.53	1.5 (1–2)	1–2	
**Physical function**				
Preoperative	54.38 ± 21.62	62.5 (50–66.25)	10–75	0.016 §
Postoperative	78.75 ± 28.25	87.5 (83.75–91.25)	10–95	
**Role limitation due to physical health**				
Preoperative	18.75 ± 29.12	0 (0–31.25)	0–75	0.025 §
Postoperative	81.25 ± 37.2	100 (87.5–100)	0–100	
**Role limitation due to emotional problems**				
Preoperative	62.5 ± 41.55	66.67 (33.333–100)	0–100	0.059 §
Postoperative	91.67 ± 15.43	100 (91.667–100)	66.67–100	
**Fatigue**				
Preoperative	48.12 ± 22.98	57.5 (27.5–65)	15–75	0.778 §
Postoperative	46.25 ± 5.18	50 (40–50)	40–50	
**Emotional wellbeing**				
Preoperative	58.5 ± 15.11	58 (51–69)	32–80	0.125 ‡
Postoperative	70 ± 8.82	72 (66–73)	56–84	
**Social functioning**				
Preoperative	64.06 ± 24.49	62.5 (50–78.125)	25–100	0.034 §
Postoperative	92.19 ± 22.1	100 (100–100)	37.5–100	
**Bodily pain**				
Preoperative	78.12 ± 25.31	88.75 (53.125–100)	45–100	0.497 §
Postoperative	85.62 ± 14.93	90 (69.375–100)	67.5–100	
**General health**				
Preoperative	38.75 ± 24.89	30 (20–57.5)	10–80	0.003 ‡
Postoperative	77.5 ± 8.86	77.5 (73.75–80)	65–95	

Abbreviations: MRC, Medical Research Council; SD, standard deviation.

§ Wilcoxon signed rank test, ^‡^ Paired t test

## Discussion

Laryngotracheal stenosis refers to the reduction in the caliber of the airway as a consequence of prolonged intubation, tracheostomy, trauma, neoplasm, autoimmune disorders, or may even be idiopathic in nature. At present, it is believed that 50% of all adult laryngotracheal stenosis is related to intubation-related injuries. 1 For cases of isolated postintubation stenosis, the gold standard of treatment remains tracheal resection and end-to-end anastomosis. 2

The diagnosis and management of tracheal stenosis pose a conundrum for clinicians as it may masquerade as a spectrum of respiratory ailments from mild wheeze to frank stridor and exertional dyspnea. 5 Patients generally present with decreased exercise tolerance due to dyspnea with stridor elicited only on exertion. Patients with a preexisting cough may report an increase in the amount of sputum with difficulty in coughing it out. The cough is often characteristic and has been described as a ‘brassy slurred cough’. These symptoms often in conjunction with intermittent wheezing episodes often lead to the erroneous diagnosis of asthma, bronchitis, or respiratory failure. Severe strictures (luminal diameter < 5 mm) manifest as inspiratory stridor at rest and are easily diagnosed while patients with mild-to-moderate tracheal obstruction may go undetected for many years. 3

Our study population consisted exclusively of patients who developed tracheal stenosis as a result of intubation-related injury. This is in keeping with the findings of Tayfun et al. and Kanlikama et al., who found that the predominant etiology of stenosis was intubation related. 6 7 However, our study differs from theirs in the indication for intubation per se, with the predominant indication in our study population being suicidal organophosphate poisoning (69%) as opposed to traffic accidents, as described by Kanlikama et al. 7 This may be attributed to the difference in the socioeconomic status of the study populations and the higher incidence of suicidal attempts in Pondicherry.

Songu et al. commented that the risk for development of tracheal stenosis increases beyond 48 hours of intubation. 8 It has been found that maximal visually observed mucosal damage occurs between 3 and 7 days. Extubation within this period usually results in complete healing; however, if endotracheal intubation is continued, mucosal damage progresses, resulting in scar formation. Further research is required to formulate guidelines for appropriate timing of tracheostomy for intubated patients to avoid cricotracheal stenoses. Our patients had been kept intubated for a mean duration of 12.5 +/− 4.58 days before they were extubated/tracheostomized.

In our series, we found that the mean length of stenosis was 1.68 +/− 1.09 cm, extending from 0.62 +/− 1.29 cm from the lower border of the cricoid as per preoperative CT scans. We estimated the mean length of the tracheal segment to be resected to be 4.75 +/− 1.41 cm. Therefore, there is an apparent loss of 3.20 +/− 1.90 cm of the normal trachea. The discrepancy between the length of stenosis and the actual length of the trachea resected can be accounted for by the low position of the tracheostoma which necessitated the excision of an apparently normal segment of trachea between the stenotic segment and the tracheostoma. The low position of the tracheostoma is attributed to the delayed clinical presentation of these patients with moderate-to-severe stridor to us, which required emergency tracheostomy as a life-saving procedure, and, hence, the tracheostoma was created with mere visualization of the closest tracheal segment possible below the lower border of the stenotic segment. In all 13 patients, tracheostoma could be created only below the level of the stenotic segment. Hence, stenoses which were previously considered short-segment have now become essentially long-segment stenoses. This puts our data at the higher end of the length recommended for resection as per previous studies. This might account for the lower rate of decannulation in our study population (61.53% as compared to 90–99% described previously). Thus, apart from the CT parameters, we have observed that the tracheostoma position serves as a major determinant for the length of resection. The extent of loss of normal trachea in addition to the stenotic segment has not been studied previously.

Postintubation tracheal stenosis remains a perplexing and difficult-to-treat condition. Though newer modalities are available for its treatment, tracheal resection and anastomosis still remain the gold standard for the management of grades III and IV stenoses.

Resection and anastomosis remain an intricate surgical procedure which require extensive preoperative evaluation and prudent patient selection. The location of the stenosis, its length, the position of the tracheostoma, the length of normal trachea available for anastomosis, the presence or absence of granulations, tracheomalacia, and suprastomal collapse of the trachea are but a few of the preoperative factors that may impact the surgical outcome. While endoscopic assessment and CT parameters should be considered in tandem during patient selection, radiological findings ought to be scrutinized with due consideration given to its low sensitivity in determining the length of the stenosis.

Though the reported incidence of risks associated with the procedure is low, they can have far-reaching consequences on the surgical and functional outcome as well as patient satisfaction. Hence, regular postoperative follow-up is mandatory to assess for restenosis, which if identified early may be managed by endoscopic dilation thus obviating the need for a revision tracheostomy. With diligent postoperative care, tracheal resection and anastomosis offer the patient a QoL that is comparable with the pre-disease one.

We found no factors affecting the success of decannulation. Among the previous studies, Ahn SH et al. reported that in patients with tracheal stenosis, those with advanced age (> 60 years) and those having a higher grade of stenosis had a significantly lower cannulation success rate. 9 In a study by Bishnoi et al., the factors affecting decannulation were lack of swallowing/secretions/cough management and the development of stridor. 10

Further research is necessary to determine the risk factors for the development of postintubation stenosis and to develop guidelines regarding the timing of tracheostomy in intubated patients. Moreover, future studies are needed to assess patient outcomes in terms of both subjective and objective parameters to provide surgeons with a comprehensive interpretation of the results of the surgery.

We encountered complications in the form of granulations in 23.07% of patients (n = 3), which is higher than that described in previous studies (4–14%), restenosis in 15.38% (n = 2), which is in line with previous data (6.2–45.6%), and no incidence of anastomotic dehiscence. Previous studies estimated the incidence of anastomotic dehiscence to be 0–46.5%. A total of 23.07% of our patients required endoscopic dilatation in comparison with 0.2 to 56.7%, according to the literature, and 15.38% could not be weaned off the T-tube, which is in agreement with the data of previous studies (3.1–14.7%). ( Table 5 )

**Table 5:** Comparison of outcomes of previous studies

STUDY	n	MEAN LENGTH RESECTED (Range/mean) (cm)	GRANULATION (%)	RESTENOSIS (%)	DEHISCENCE (%)	DURATION OF FOLLOW-UP (years)	DECANNULATIONRATE (%)	POSTOPERATIVE DILATION (%)	T-TUBE (%)	REVISION SURGERY (%)	MORTALITY (%)
Pearson et al. (1971) 3	37	3–5		16.21			89.19			16.21	5.40
Laccourreye et al. (1996) 11	32			6.20		5			3.1	3.1	
El-Fattah 12	12	2–4				10	91	16			
Wright (2004) 13	900	3.3	8.6	45.6	45.6		95	0.2	4	1.7	1.2
Bibas et al. (2014) 14	94	2.9	4	16	1		96.8	12	6.38	1.06	0
H Elsayed (2016) 15	30		8.8				96.7	56.7	Stent 13.3	3.33	3.33
Mohsen et al. (2018) 16	52	4.378	–	13.4	0	10	86.5	7.69			0
Piazza et al. (2014) 17	137	2.7	14	38	47.6		99				< 1
Mutrie et al. (2011) 18	105	2.7	–	17	1	3	98	17		3	1
D'Andrilli et al. (2016) 19	109	3.4	–	7.4	0.9	4.3	94.5			0	0
Kanlikama et al. (2018) 7	34		(8.8)	32.4	(5.9)	0.5	(91.1)	(8.8)	14.7	14.70	(2.9)
Present study	13	4.75	23.07	15.38	–	0.25	61.53	23.7	15.8	–	0

The deviations in our data from those described in prior studies might be attributed to our limited sample size and short duration of follow-up. None of our patients encountered any non-anastomotic complications, including wound infection, dysphagia, aspiration, hoarseness, laryngeal nerve palsy, or pneumonia.

Overall, the patients showed significant improvement in the QoL and dyspnea severity after treatment with minimal complications. In comparison, Pullens et al. found that the QoL of children in terms of physical functioning, role functioning: emotional/behavior, and general health perceptions were significantly worse in patients with tracheal stenosis as compared to the normal population. 20

Tayfun et al. concluded that all the parameters considered under SF-36, except ‘role emotional’, were found to be statistically significant ( *p* < 0.01) in comparison to preoperative scores. 6 This is in direct juxtaposition with our findings, which showed significant postoperative improvement only in the domains of physical function and general health. In both our studies, there was a significant improvement in MRC dyspnea scores postoperatively.

## Limitations of the Study

The reliability of this data is influenced by our small sample size and short duration of follow-up. While we have sought to assess the subjective outcomes through patient-administered questionnaires, it is beyond the scope of this study to assess the functional outcomes of surgery using objective tests, such as the treadmill test, pulmonary function tests, Grade of Hoarseness, Roughness, Breathiness, Asthenia, Strain (GRBAS) scale, and Functional Endoscopic Evaluation of Swallowing (FEES), which would provide more comprehensive data regarding postoperative QoL.

## Conclusion

Diligent placement of tracheostomy in an emergency setting with respect to the stenotic segment plays a pivotal role in minimizing the LoTR, which is of paramount importance for the success rate. From our study, we conclude that it is not only essential to calculate the preoperative stenotic segment of the trachea but also to include the normal trachea that would be lost on account of including the previous tracheostoma as this serves as a key determinant while planning resection.

## Declarations

 
