# Supplementary material for: Site of Tracheostomy and Its Influence on The Surgical Outcome and Quality of Life After Tracheal Resection and Anastomosis in Patients with Tracheal Stenosis
Source: Int Arch Otorhinolaryngol. 2024 Jan 4;28(1):e22–9. doi: 10.1055/s-0043-1776702 (PMC10843922; doi:10.1055/s-0043-1776702)
Supplement: Supplementary file 1 — Supplementary Material [file 10-1055-s-0043-1776702-s2022031232or.pdf]

Supplementary Material File 1 RAND 36-Item Short Form Health Survey Instrument.

கேள்வித்தாள்

1. பொதுவாக உங்கள் உடல் ஆரோக்கியம் குறித்து எவ்வாறு கருதுகிறீர்கள்?	
சிறப்பாக உள்ளது	1
மிகவும் நன்றாக உள்ளது	2
நலமாக உள்ளது	3
மோசமாக உள்ளது	4
மிகவும் மோசமாக உள்ளது	5

2. கடந்த ஆண்டோடு ஒப்பிடுகையில், தங்கள் வாழ்க்கைத் தரம் தற்போது எவ்வாறு உள்ளது.?	
கடந்த ஆண்டை விட, மிகவும் சிறப்பாக உள்ளது	1
கடந்த ஆண்டை விட, ஓரளவு சிறப்பாக உள்ளது	2
கடந்த ஆண்டைபோல உள்ளது	3
கடந்த ஆண்டைவிட, சிறிது மோசமாக உள்ளது	4
கடந்த ஆண்டைவிட, மிகவும் மோசமாக உள்ளது	5

பின்வரும் கேள்விகள் தங்களின் தினசரி செயல்பாடுகளை குறித்து வருபவை, தங்களின் அன்றாட செயல்பாடுகள் தற்போதைய உடல்

நிலையினால் பாதிக்கப்பட்டுள்ளதா? ஆம் என்றால், எவ்வளவு?

	ஆம் பெரிதும் பாதிக்கப்பட்டுள்ளது	ஆம் சிறிதளவு பாதிக்கப்பட்டுள்ளது	இல்லை பாதிப்பு ஏதும் இல்லை
3. தீவிரமான செயல்பாடுகள், ஓடுதல், அதிக எடை தூக்குதல், விறுவிறுப்பான விளையாட்டுப் போட்டிகளில் பங்கேற்றல், கடினமான வேலை செய்தல்.	1	2	3

 © 2024. Fundação Otorrinolaringologia. All rights reserved.

4. மிதமான செயல்பாடுகள்,<br><br>மேசையை நகர்த்தல், பந்து எறிதல், வீடுபெருக்குதல்.	1	2	3
5. எளிமையான பொருள்கள் தூக்குதல் அல்லது சுமந்து செல்லுதல்.	1	2	3
6. பல மாடிபடிகள் ஏறுதல்.	1	2	3
7. ஓர் மாடிபடிஏறுதல்.	1	2	3
8. வளைதல், முட்டிபோடுதல், குனித்தல்	1	2	3
9. ஒரு மைலுக்கு மேல் நடத்தல்.	1	2	3
10. பல நூறு அடிகள் நடத்தல்.	1	2	3
11.குறைந்தது நூறு அடிகளுக்கும் குறைவாகவே நடத்தல்.	1	2	3
12. தானே குளிக்கவும், உடை உடுத்தவும் செய்தல்.	1	2	3

கடந்த நான்கு வாரங்களில், தங்களுக்கு உடல் ரீதியாக பணியிடத்தில் அல்லது அன்றாட செயல்களில் கீழ்க்காணும் பிரச்சினைகள் ஏதேனும் இருந்ததா?

ஒவ்வொரு வினாவிற்கு சரியான பதிலை வட்டமிடுக

	ஆம்	இல்லை
13. பணியில் அல்லது மற்ற செயல்களில் செலவிடும் நேரம் குறைக்க வேண்டிய நிலை ஏற்பட்டது.	1	2
14. தாங்கள் விரும்பியதை விட குறைந்த அளவே செயல்கள் நிறைவேற்றப்பட்டது.	1	2

 © 2024. Fundação Otorrinolaringologia. All rights reserved.

15. தாங்கள் செய்த சில வகை பணிகள் அல்லது சில செயல்பாடுகள் தற்போது பாதிக்கப்பட்டுள்ளது .	1	2
16. தாங்கள் செய்யக் கூடிய பணி அல்லது மற்ற செயல்கள் செய்வதற்கு இப்பொழுது சிரமமாக இருந்தது. (உதாரணம் கூடுதல் முயற்சி தேவைப்படுகிறது).	1	2

கடந்த நான்கு வாரங்களில், தங்களுக்கு உணர்வு பூர்வமாக பணியிடத்தில் அல்லது அன்றாட செயல்களில் கீழ்க்காணும் பிரச்சினைகள் நடந்ததா? (உதாரணம் சோர்வு அல்லது படபடப்பு)

	ஆம்	இல்லை
17. பணியில் அல்லது மற்ற செயல்களில் செலவிடும் நேரம் குறைக்க வேண்டிய நிலை ஏற்பட்டது.	1	2
18. தாங்கள் விரும்பியதை விட குறைந்த அளவே செயல்கள் நிறைவேற்றப்பட்டது.	1	2
19.எப்போதும் செய்யும்பணி அல்லது மற்ற செயல்களில் முன்பைக் காட்டிலும் கவனமாக செய்ய இயலவில்லை.	1	2

20. கடந்த நான்கு வாரங்களில், எந்த அளவிற்கு தங்களுக்கு உடல் ரீதியாகவோ அல்லது உணர்வு பூர்வமாகவோ பிரச்சனைகள் உங்கள் சாதாரண சமூக பழக்கவழக்கங்களை குடும்பத்தினருடனும், நண்பர்களுடனும், உறவினர்களுடனும் எதிர்கொள்ள நேரிட்டது?.

(ஒரு எண்ணை வட்டமிடு)

இல்லவே இல்லை 1

சிறிதளவு 2

மிதமாக 3

ஓர் அளவிற்கு 4

மிகவும் அதிகமாக 5

 © 2024. Fundação Otorrinolaringologia. All rights reserved.

21. கடந்த நான்கு வாரங்களில், எவ்வளவு உடல் ரீதியான வலியினால் வருந்தினீர்கள்?

எதுவும் இல்லை	1
மிகவும் லேசாக	2
லேசாக	3
மிதமாக	4
அதிகமாக	5
மிகவும் அதிகமாக	6

22. கடந்த நான்கு வாரங்களில், இவ்வலியினால் உங்கள் அன்றாட பணிகள் எவ்வளவு பாதிப்புக்கு உள்ளாகியது? (உதாரணம் வீட்டு வேலைகள் மற்றும் வெளிப்புற செயல்பாடுகள் உட்பட)

இல்லவே இல்லை	1
சிறிது அளவிற்கு	2
மிதமான அளவிற்கு	3
ஓர் அளவிற்கு	4
மிகவும் அதிகமாக	5

கடந்த நான்கு வாரங்களில், பின்வரும் கேள்விகளுக்கு, தங்களுக்கு நடந்த நிகழ்வுகளையும் தங்கள் உணர்வுகளையும் எவ்வாறு எண்ணுகிறீர்கள்? அவ்வாறான எண்ணங்கள் எவ்வளவு முறை தோன்றியது? தங்கள் எண்ணத்தில் சரியாக குறிக்கும் ஓர் பதிலை வட்டமிடுக.

	எந்நேரமும்	பெரும்பாலான நேரம்	ரொம்ப நேரம்	இல நேரங்களில்	எப்போதாவது மட்டும்	எப்போதும் இல்லை
23. முழு ஆற்றலுடன் இருப்பதாக உணர்ந்தீர்களா?	1	2	3	4	5	6
24. எப்போதாவது பதட்டமாக இருந்திருக்கிறீர்களா ?	1	2	3	4	5	6

 © 2024. Fundação Otorrinolaringologia. All rights reserved.

25. எப்போதாவது தாங்கள் மிகவும் தளர்ந்து போய் இருந்த நேரத்தில் எவ்வித புத்துணர்ச்சி முற்சிகளும் பயனளிக்காமல் இருந்ததாக உணர்கிறீர்களா?	1	2	3	4	5	6
26. அமைதியாகவும் நிம்மதியாகவும் உணர்ந்தீர்களா?	1	2	3	4	5	6
27. அதிக ஆற்றல் உள்ளதாக உணர்ந்தீர்களா?	1	2	3	4	5	6
28. மனமுடைந்து காணப்பட்டீர்களா?	1	2	3	4	5	6
29. தோய்வடைந்தாக உணர்ந்தீர்களா?	1	2	3	4	5	6
30. சந்தோஷமான மனிதராக உணர்ந்தீர்களா?	1	2	3	4	5	6
31. சோர்வடைந்தாக உணர்ந்தீர்களா?	1	2	3	4	5	6

32. கடந்த நான்கு வாரங்களில் எவ்வளவு நேரம் உங்கள் உடல் உபாதைகளால் அல்லது மன உளைச்சல்களினால் தங்கள் சமூகபழக்கவழக்கங்கள் பாதிக்கப்பட்டது?. (எ.கா நண்பர்கள், உறவினர் சந்திப்பது போன்றவை)

எந்நேரமும் 1

நிறையநேரம் 2

சிலநேரங்களில்மட்டும் 3

எப்போதாவதுமட்டும் 4

எப்பொழுதும்இல்லை 5

பின்வருபவகைகளைப் படித்து சரியா? தவறா? என்றுதெரிவிக்கவும்.

 © 2024. Fundação Otorrinolaringologia. All rights reserved.

	உறுதியாக சரி	பெரும்பாலும் சரி	தெரியவில்லை	பெரும்பாலும் தவறு	உறுதியாக தவறு
33. நான் மற்றவர்களைக் காட்டிலும் எளிதாக சோர்ந்து போகிறேன்.	1	2	3	4	5
34. நான் மற்றவர்களைப் போல் ஆரோக்கியமாக எண்ணுகிறேன்.	1	2	3	4	5
35. நான் என் உடல் ஆரோக்கியம் மோசமாகுமோ என்று எண்ணுகிறேன்.	1	2	3	4	5
36. என் உடல் ஆரோக்கியம் மிகவும் சிறப்பாக உள்ளது.	1	2	3	4	5

 © 2024. Fundação Otorrinolaringologia. All rights reserved.
